# Maternal hypothalamic-pituitary-adrenal axis response to foraging uncertainty: A model of individual *vs*. social allostasis and the "Superorganism Hypothesis"

**DOI:** 10.1371/journal.pone.0184340

**Published:** 2017-09-07

**Authors:** Jeremy D. Coplan, Nishant K. Gupta, Asif Karim, Anna Rozenboym, Eric L. P. Smith, John G. Kral, Leonard A. Rosenblum

**Affiliations:** 1 Department of Psychiatry and Behavioral Sciences, Biological Science Unit, State University of New York (SUNY) Downstate Medical Center, Brooklyn, New York, United States of America; 2 College of Medicine, SUNY Downstate Medical Center, Brooklyn, New York, United States of America; 3 Kingsborough Community College, Brooklyn, New York, United States of America; 4 Departments of Internal Medicine and Surgery, SUNY Downstate Medical Center, Brooklyn, New York, United States of America; Oregon Health and Science University, UNITED STATES

## Abstract

**Introduction:**

Food insecurity is a major global contributor to developmental origins of adult disease. The allostatic load of maternal food uncertainty from variable foraging demand (VFD) activates corticotropin-releasing factor (CRF) without eliciting hypothalamic-pituitary-adrenal (HPA) activation measured on a group level. Individual homeostatic adaptations of the HPA axis may subserve second-order homeostasis, a process we provisionally term “social allostasis.” We postulate that maternal food insecurity induces a “superorganism” state through coordination of individual HPA axis response.

**Methods:**

Twenty-four socially-housed bonnet macaque maternal-infant dyads were exposed to 16 weeks of alternating two-week epochs of low or high foraging demand shown to compromise normative maternal-infant rearing. Cerebrospinal fluid (CSF) CRF concentrations and plasma cortisol were measured pre- and post-VFD. Dyadic distance was measured, and blinded observers performed pre-VFD social ranking assessments.

**Results:**

Despite marked *individual* cortisol responses (mean change = 20%) there was an absence of maternal HPA axis *group* mean response to VFD (0%). Whereas *individual* CSF CRF concentrations change = 56%, *group* mean did increase 25% (p = 0.002). Our "dyadic vulnerability" index (low infant weight, low maternal weight, subordinate maternal social status and reduced dyadic distance) predicted maternal cortisol decreases (p < 0.0001) whereas relatively “advantaged” dyads exhibited maternal cortisol increases in response to VFD exposure.

**Comment:**

In response to a chronic stressor, relative dyadic vulnerability plays a significant role in determining the directionality and magnitude of individual maternal HPA axis responses in the service of maintaining a “superorganism” version of HPA axis homeostasis, provisionally termed “social allostasis.”

## Introduction

Allostasis is a concept developed by McEwen and Stellar which refers to the process whereby the organism achieves homeostasis through physiologic or behavioral changes in response to an allostatic load or chronic stressful conditions [1–4]. Allostatic homeostasis may occur through multiple processes such as alterations in the hypothalamic-pituitary-adrenal (HPA) axis, autonomic nervous system, or cytokines [5]. Although allostasis is generally viewed as adaptive in the short-term, prolonged allostasis in the face of chronic allostatic load can exert deleterious long-term effects on health downstream from the onset of the initial environmental insult or psychosocial stressor [6]. In humans, the persistence of cumulative stress eventually drives maladaptive homeostatic responses, which in turn predict poor health outcomes, further driving an allostatic load [7]. Chronic stress has been hypothesized to initiate a cascade of glucocorticoid suppression, cytokine-mediated hyperimmunity, insulin resistance and ultimately endothelial inflammation [8], accelerating the development of cardiovascular disease and metabolic obesity [9]. Chronic stress also drives inflammation and carcinogenesis [7].

Adverse early life events may trigger adaptive-oriented responses within the nervous, endocrine, and immune systems in children, thereby setting the stage for susceptibility to the development of adult anxiety and mood disorders [10, 11]. Perhaps the most persistent and durable postnatal homeostatic alterations are those induced by allostasis occurring early in neurodevelopment. The infant phase of postnatal neurodevelopment represents a period of extensive neuronal migration, neuronal dendrification and synaptogenesis and subsequent pruning of targeted projection sites [12], thus rendering the infant particularly vulnerable to neurotoxicity in general during neurodevelopment [13]. Prolonged exposure of socially-housed nursing mothers to unpredictable foraging conditions [termed variable foraging demand (VFD)] is persistently evident in offspring physiology in the form of continued allostatic overload modifications despite stressor cessation many years previously [14].

The VFD procedure involves 16 repeated, abrupt and, for nonhuman primate mothers, putatively unpredictable shifts from easy [low foraging demand (LFD)] to hard [hard foraging demand (HFD)] to easy foraging in two-week blocks [15]. The repeated change of foraging demand overwhelms maternal coping capacity and appears to induce a form of emotional separation between mother and infant [16]. As a consequence, we hypothesized that the mother becomes “emotionally unavailable” to her infant [17]. The VFD model has been substantiated by numerous studies. In particular, we have previously shown that macaques reared under HFD only conditions exhibited no significant difference in CRF concentrations as compared to the LFD group. However, the VFD group exhibited significant and persistent elevation of CSF CRF concentrations [14]. As a consequence of VFD procedure, we hypothesized that the mother becomes emotionally unavailable to her infant [17]. Although studies on offspring are numerous, fewer may have focused on the neurobiology underlying maternal emotional unavailability [18]. Given that stress is transmitted from mother to infant [19], interventions intended to prevent the cycle of offspring compromise necessitate maternal relief from allostatic overload and compromise of dyadic ventral-ventral contact [20].

Mothers exposed to the VFD stressor exhibit an unexpectedly strong influence (> 80% of the variance) of social rank on maternal-infant proximity—relative increases in dyadic distance are observed in socially dominant mothers versus increased dyadic proximity in socially subordinate subjects [21]. Given the absence of a relationship between social rank and dyadic distance in VFD-unexposed conditions, hyper-attentiveness to the social hierarchy evidently diverts maternal focus from the essential task of contingent responsivity implicit in normative rearing [21]. Mothers undergoing VFD exposure exhibit an increase in cerebrospinal fluid (CSF) corticotropin-releasing-factor (CRF) concentrations, which is mirrored by synchronized activation of central CRF in their infants [19]. A potential dissociation was observed with marked activation of the maternal CRF system in response to an experimentally induced allostatic load [18] while activation of the maternal HPA axis, either in relation to the pre-VFD level or in group mean comparisons to controls, was notably absent. The dissociation may, in part, be related to the distinction between the HPA axis and extrahypothalamic CRF release [22]. However, the absence of any HPA axis change warrants scrutiny. We previously noted that VFD-reared macaques exhibit elevated levels of CSF CRF concentrations with paradoxically decreased basal CSF cortisol levels [2]. Recently, a pilot study found that juvenile CSF CRF in male macaques, associated with the VFD rearing conditions, correlated directly with adult monocytic glucocorticoid receptor (GR) expression volatility during an acute stressor [23] (see Appendix for further details for CRF/HPA axis interaction).

Although allostasis has been well validated as a concept, biological markers can help in its quantification. Schulkin and colleagues identified CRF activation as a critical physiological mediator of allostatic response [24]. Quantification of allostatic stress [25] typically analyzes differences in group means comparing the allostatic condition versus controls In the absence of group mean changes, variation in individual responses should be considered [2]. In Grafen's hypothesis of ‘Darwinian driven superorganism adaptations,’ the agents are deemed to be individual organisms [26]. This view is expected because individuals are usually understood as the agents of adaptations in biology. However, the hypothesis to be tested, using the Grafen hypothesis [27], is to test “whether, and in what circumstances, whole groups can legitimately be considered as adaptive units, or ‘maximizing agents’.”

In this context, nonhuman primate models of allostasis may complement human studies as follows: 1) the opportunity to study allostatic stress in a randomly-assigned controlled design in the context of complex social nonhuman primate female groups and their infants, 2) emphasis away from group mean changes with a focus on individual changes that correlate with specialized social adaptations to the allostatic load, and 3) the opportunity to examine the impact of maternal allostasis in the context of the mother-infant relationship where specific features of dyadic vulnerability bear homology to humans. Homeostatic adaptations to chronic stress may not only pertain to the individual but also to the social group functioning as a single integrated and coordinated entity, a phenomenon we provisionally term “social allostasis.” The concept is not novel with respect to female reproductive function, with synchrony observed in the human menstruation cycle when housed socially, although this area is not without controversy [28]. Synchronized gonadal hormone patterns have been described in female macaques [29].

In the current study, basal plasma cortisol levels in a cohort of socially housed nursing bonnet macaques were evaluated before and at the cessation of VFD exposure. These subjects exhibited an overall increase in maternal and infant CSF CRF in response to the VFD stressor [14] providing strong evidence of dyadic allostasis. Based on our hypothesis that food insecurity would, via the HPA axis, induce neurometabolic alterations [30], we re-examined cortisol data from the dyads in a previous report on CSF CRF concentrations [19, 31]. We hypothesized that “social allostasis” in response to VFD would induce significant *individual* HPA axis alterations that were bidirectional, therefore maintaining group mean cortisol levels within a narrow range—a “supraorganism limitation.” We hypothesized that a putative profile of dyadic vulnerability would be associated with relatively lower levels of plasma cortisol. By contrast, relative hypercortisolism would be associated with a relatively robust dyadic environment. The ultimate result of these divergent phenotypic responses may be evident only as minimal change in group means, yet represent relatively pronounced individual differences in maternal HPA axis response to VFD exposure.

## Methods

### Ethics statement

All animal work was conducted at the Nonhuman Primate Facility of the State University of New York Downstate Medical Center with permission from its Institutional Animal Care and Use Committee (IACUC), in accordance with the recommendations of the Weatherall report, “The use of non-human primates in research.” The welfare of the animals conformed to the requirements of *National Institutes of Mental Health (NIMH)*.

### Subjects

Twenty-four mother-infant bonnet macaque dyads served as subjects, with all subjects born and reared in social groups within SUNY Downstate Nonhuman Primate facility. These subjects have been reported upon previously [19, 21] but our analyses at the time failed to address the potential significance of the absence of maternal HPA alterations despite significant activation of maternal central CRF systems. All mother-infant dyads were exposed to variable foraging demand (VFD) conditions [15].

**Fig 1:**
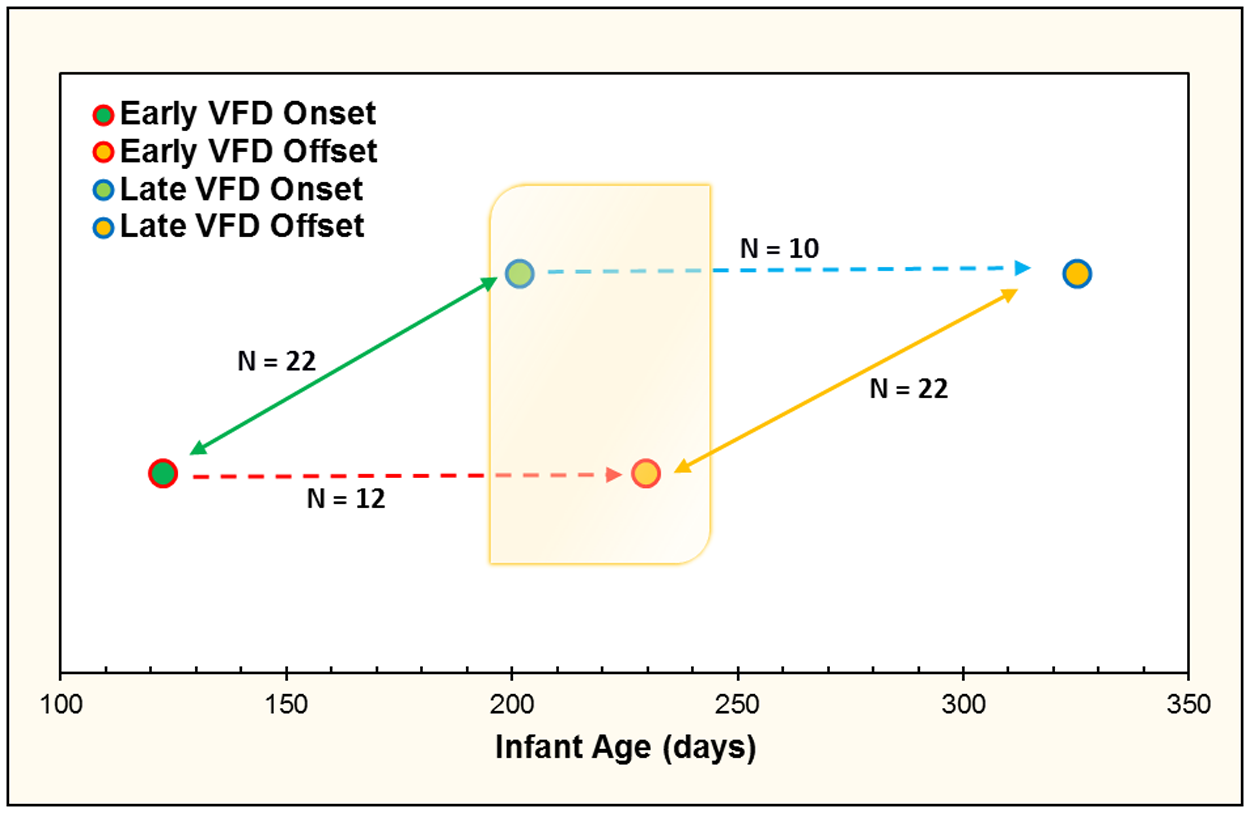
Representation of study design with staggered onset of VFD exposure based on infant age allowing for cross-sectional data analysis. The shaded orange box represents the overlapping period of VFD rearing procedure that allowed for cross-sectional analyses, where the early onset group is entering the final phase of VFD, and late onset group is entering the initial phase of VFD. Blue and red dashed lines represent 16-week VFD phase. Bidirectional green and yellow lines represent VFD onset and offset for both groups, respectively. Mean infant ages at each interval follow: Early Onset [mean (SD) = 122.60 (37.70 days), N = 12]; Early Offset [229.70 (39.80)]; Late Onset [201.60 (22.72), N = 10]; Late Offset [325.20 (22.72)].

In the current study, we used the first two pens of four pens exposed to VFD as the source of our “post-VFD” data which could then be compared in a cross-sectional manner to the second two pens exposed to VFD which served as a “non-VFD” baseline prior to their exposure to VFD (Fig 1) [21, 32]. The range of infant age at the time of VFD onset [mean (SD) = 158.50 (50.90) days, N = 22] allowed us to reasonably compare data gathered from the offset of VFD in the early group and onset of VFD in the late group, as per our staggered design (Fig 1). Mean infant ages at each interval was as follows: Early Onset [mean (SD) = 122.60 (37.70 days), N = 12]; Early Offset [229.70 (39.80)]; Late Onset [201.60 (22.72), N = 10]; Late Offset [325.20 (22.72)]. All four pens were used for their pre-VFD and post-VFD values for longitudinal comparison. HPA axis data on two subjects were not available, leading to an N of 22 available for analysis. The range of infant age at the time of VFD onset [mean (SD) = 158.50 (50.90) days, N = 22] allowed us to reasonably compare data gathered from the offset of VFD in the early group and onset of VFD in the late group, as per our staggered design (Fig 1). Mean infant ages at each interval was as follows: Early Onset [mean (SD) = 122.60 (37.70 days), N = 12]; Early Offset [229.70 (39.80)]; Late Onset [201.60 (22.72), N = 10]; Late Offset [325.20 (22.72)]. A trend difference for infant age in the cross-sectional comparison [t-value = -2.41, p = 0.06] was noted and the latter was therefore used as a covariate [21]. Maternal and infant weights were recorded immediately before induction of VFD. Stability of maternal weight and normative infant weight gain were maintained in all subjects across the duration of the VFD procedure [14].

### Housing

All subjects were socially housed in one of four pens and observed in tile-walled pens approximately 6 x 12 x 7 feet high with mesh ceilings, two levels of shelving, and two automatic watering spouts. Each pen was fronted by two large one-way observation screens, through which all observations were made. Fluorescent lighting was set to a 12:12 schedule.

The twenty-four mother-infant dyads were distributed across four pens of 4–7 dyads, with group formation taking place for at least 8 weeks prior to VFD inception, allowing for group stabilization prior to the onset of experimental conditions. Adult males were removed after female pregnancy was documented.

### VFD rearing

For the VFD rearing exposure [15], mothers were confronted with an environment in which an adequate amount of food was always available yet the amount of time and effort necessary to obtain daily rations leading to a perception of foraging uncertainty, with persistent psychobiological effects observed in grown offspring [33]. The maternal food procurement schedule consisted of alternating blocks of 2-weeks in which food was easy to find (Low Foraging Demand; LFD), and 2-weeks in which food procurement was difficult, involving more time and effort (High Foraging Demand; HFD). Beginning with LFD, a total of four, 2-week periods of LFD and four alternating 2-week periods of HFD comprised the 16-week VFD experimental period.

“Foraging carts” with multiple apertures on both sides of the cart were used to vary foraging demand whereby a) food could either be buried in wood chips (HFD), requiring animals to search for and retrieve food or b) food left freely exposed in the carts’ containers (LFD). Further details of the “Foraging Cart” and the VFD procedure can be found in Andrews and Rosenblum [34].

### Social rank

Mothers were assessed for hierarchical status during the final LFD phase of the 16-week VFD cycle. Assessment of social rank was independently conducted by two experienced observers. Only one disagreement was recorded (one mid-rank difference) in all 24 subjects.

Social rank of each mother was scored by a rank of most dominant (accorded a score of 1) to most subordinate (accorded a score of 4–7 depending on the group). Scoring was assessed during the final 2-week phase of the 16-week VFD cycle. This final 2-week phase always ends with an HFD cycle. This time point corresponded with the ad libitum state of the control group during these final two weeks. While social rank was only formally measured during this final phase subjects were monitored throughout the study period, and as per protocol, the principal investigator (PI) was to be notified of any behaviors or incidents indicating social upheaval of the group (e.g. physical harm to subjects, wounding). At no point during the experimental procedure was the PI notified of such incidents.

Maternal social rank was assessed by two independent blinded observers who scored agonistic encounters between the maternal subjects until every possible pairing has been observed and assessed. Observers were trained to observe for typical dominant and subordinate behaviors. Subordinate behaviors include: *Displacement*: the subordinate subject flees from the dominant subject without the presence of threat or aggression; *Lip smack/crouch/present*: the subordinate subject exhibits rapid lip opening and closing with repeated tongue extrusion, lowering of chest or presenting rump in the direction of the dominant subject. Dominant behaviors include: *Threat*: the dominant subject displays an aggressive facial expression towards the subordinate subject, identified by open mouth, bared teeth and retracted ears; *Aggressive Chase*: the dominant subject chases the subordinate subject causing it to flee, without physical aggression; *Mounting*: dominant subject mounts the subordinate without sexual intent [21].

Social rank was then tabulated by each of the observers for each subject. Rank ambiguity presented in the mid-ranking subjects. To address this situation the observer was required to assess three additional agonistic encounters between subjects with rank ambiguity. Score provided by the observers was with a kappa score greater than 0.95.

Since the inception of this breeding colony in the 1960s, more than 1200 births and infant developments have been observed in similar settings [35]. The VFD model has been used since 1961 and is well established in the literature. Observations since then have suggested that established hierarchies are stable over the course of at least the first postnatal year. The groups in this study were established prior to the onset of any pregnancies and in most cases, had been part of the same social structure for a minimum of 8 months prior to the birth of the first infant and prior to introduction of male during the harem phase [15]. Males are removed from the group after female pregnancy is documented. VFD inception does not begin for at least another 8 weeks. Once this group structure forms, it has been known to remain stable even through multiple generations [36, 37]. In fact, previous studies in our own lab have revealed social rank of VFD reared dyads has serves as a significant predictor of maternal-infant proximity [21]. In addition, all members of each social group have been born and bred in our laboratory and are well adapted to basic laboratory procedures, largely eliminating environmental perturbations that may have shaken group stability over the course of the study period. Furthermore, rank stability has been consistently noted in several studies in rhesus macaque (*Macaca mulatta*) models unless social rank is experimentally manipulated [38, 39].

### Maternal-infant proximity (Dyadic-distance)

During daily behavioral sessions, each mother-infant dyad was observed for 20 seconds in a random sequence on five serial observation periods. Mother and infants were identified by distinguishing physical features by experienced primatologists. Dyadic-distance was scored during each observation on a scale of 1–5 based upon initial assessment. A score of 1 was given when mother and infant were in direct “ventral-ventral” contact. A score of 5 was given when the dyad was at a maximum distance in the pen, as permitted by the pen dimensions. A score of two was awarded when the mother and the infant were less than one meter apart. A score of three was awarded if maternal-infant distance was greater than one meter. A score of four was awarded when maternal-infant distance was in between a three and a five-meter distance. Mother-infant dyadic-distance was aggregated over two weeks during the final LFD phase and final HFD phase of the 16-week VFD cycle.

### Biological samples

Peripheral blood and cisternal CSF samples were obtained prior to the onset and during the last week of the VFD cycle. Sampling was performed between 11:00 AM to noon for all subjects. Mothers were rapidly anesthetized in the squeeze cage and as the mothers underwent anesthetic sedation, infants were administered ketamine (in rapid succession). Once the mothers’ CSF and bloods had been drawn, the infants had CSF and blood samples immediately thereafter. Approximately 10cc of blood was drawn from the saphenous vein, and three cc’s of CSF were drawn at each sample (for details of CSF sampling procedure, see Scharf et al. [40]). Capture stress was minimized in this procedure and timing of anesthetization meant there was no separation of mother-infant dyads at any time. No biological samples were taken during the VFD period *per se* in order not to perturb the VFD-experimental process.

### Laboratory measures

Plasma cortisol was determined by radioimmunoassay at the Clinical Pathology Laboratory of SUNY Downstate Medical Center, an accredited laboratory at a teaching hospital. All plasma cortisol samples were run in the same assay to eliminate inter-assay variability.

Maternal CSF CRF concentrations were analyzed by radioimmunoassay according to Altemus and colleagues [41]. All samples were measured in one assay to eliminate inter-assay variability. As per historical values, the assay has a sensitivity (90% binding) of 20pg/ml and an intraassay coefficient that ranges from 6.8% to 13.2% [42]. The present assay was run in proximity to the time of the referenced assays.

### Statistical methods

Key variables were tested for normality of distribution using the Kolmogorov-Smirnov test and data inspected for outliers. Firstly, we ran a General Linear Model 2X2 ANOVA-RM using plasma cortisol and CSF CRF concentrations (scaled to cortisol range by dividing by 10) as the first dependent measure and pre-VFD and post-VFD as the second dependent measure. Posthoc paired t-tests compared pre- to post-VFD maternal cortisol levels seeking to determine whether despite significant increases in maternal CSF CRF concentrations [18] effects for HPA axis function would not be observed. An absence of significant reduction in variance across the VFD duration was examined to rule out a “regression to the mean” effect [43]. A pre- versus post-VFD repeated measures analysis was then repeated while controlling for potential pen (N = 4) effects as a categorical variable using a general linear model (GLM) (Statistica 12).

#### Ratio determinations

Identical computations were performed for plasma cortisol and CSF CRF concentrations. The numerator would represent the magnitude of group change divided by the pre-VFD exposure baseline in percentage terms whereas the denominator would account for the magnitude in absolute percentage terms of within-subject (individual) change relative to baseline. A relatively high ratio would reflect relatively less contribution of individual versus group mean differences. A relatively low ratio would reflect a relatively greater contribution of individual versus group mean differences. The results were entered into a 2x2 table (Table 1) and tested using non-parametric statistics, as follows:

**Table 1:** Group mean vs. individual change for CRF and cortisol.

% Δ cortisol group mean/pre-VFD	% Δ CRF group mean/pre-VFD
% Δ cortisol individual change/pre-VFD	% Δ CRF individual change/pre-VFD

#### Correlational analyses

Pearson’s correlations were performed on pre- versus post-VFD cortisol, pre-VFD versus Δ cortisol and then Δ versus post-VFD cortisol. The issue we interrogated was to ascertain to what degree Δ cortisol was determined by pre-VFD cortisol and whether post-VFD cortisol was determined by pre-VFD cortisol and/or Δ cortisol. Such analyses would provide information into the origins of Δ and post-cortisol. The same analyses were performed for CSF CRF concentrations.

#### Z-score analyses

In order to further demonstrate the specificity of “social allostasis” effects to the HPA axis *vis-a-vis* the central CRF system, Δ cortisol was contrasted to Δ CRF after both variables had been converted to standardized “z-scores.” Conversion to z-scores permitted simultaneous incorporation of cortisol and CRF into a repeated measures design using a GLM. A major focus of the comparative analyses was to determine whether putative vulnerability factors for individual maternal-infant dyads resulted in maternal plasma cortisol decreases upon VFD exposure whereas relatively advantaged dyads would account for relative increases in cortisol. The latter scenario would ultimately result in minimal group mean change (and represent a form of homeostasis through social redistribution of HPA axis activity under conditions of allostatic overload). These Z Δ plasma cortisol correlations were compared through the GLM-RM analysis to the Z Δ CSF CRF correlations. Predictor variables were predicted not to correlate with the latter. The putative predictor variables were individually entered to identify parameters reflective of dyadic vulnerability—maternal factors (age, weight, social rank, pre-VFD cortisol and CSF CRF concentrations), a maternal-infant factor (dyadic distance during the final HFD phase). Infant factors included age and weight at VFD onset and infant sex. A GLM was then run using successive putative variables reflective of dyadic vulnerability as the predictor variable and the Z-scores of Δ cortisol and Δ CRF as two repeated measures. A repeated measures x predictor variable interactive effect would demonstrate divergent roles for the HPA axis and central CRF system in relation to that specific variable.

#### Cluster analysis and “dyadic vulnerability index”

A cluster analysis and examination of Euclidean distances was then performed for variables that had been demonstrated to be associated with Δ cortisol. Based on the cluster analysis, "core" vulnerability factors, excluding HPA axis measures, would be identified, preferably using a post hoc Euclidian Linkage Units cut-off, and each of the vulnerability variables identified were converted to standardized z-scores. The sum of the identified z-scores, each transformed, if necessary, to directly reflect magnitude of vulnerability, created a "dyadic vulnerability" index. Infant sex was used as a categorical variable in a GLM with the index used as the predictor variable and Δ cortisol response to VFD exposure as the dependent variable in order to support the hypothesis of divergent HPA axis responses as a function of relative dyadic vulnerability.

#### Controlling for non-specific time effects

To demonstrate that HPA axis effects were specifically due to VFD exposure and not a non-specific effect of “Time," in a cross-sectional analysis, VFD exposed mothers were predicted to differ from non-exposed subjects. We examined the relationship of post-VFD maternal cortisol obtained from “early VFD” (N = 12) versus pre-VFD cortisol taken from “late VFD” exposed mothers (N = 10). The relationship of plasma cortisol to maternal body weight (this variable was selected based on the previous Δ z plasma cortisol analysis [18]) was computed using a GLM with maternal VFD exposure as the categorical variable, plasma cortisol as the dependent variable, maternal body mass as the predictor variable and infant age as a covariate.

Probability was set at p ≤ 0.05, two tailed without correction for multiple comparisons, given the exploratory nature of the analysis.

## Results

Normality of distribution was satisfied for key variables, and no outliers were noted. Twenty-two mother-infant dyads had a full complement of plasma cortisol and CSF CRF measures (Table 2).

**Table 2:** Maternal and infant predictor variables

Variable	N	Mean	Std. Dev.	Minimum	Maximum
Maternal Age (years)	22	8.5	3	4.5	13.8
Maternal Wt. (kg.)	22	4.94	0.95	3.18	6.20
VFD Onset (days pp) *	21	158	52	64	237
Infant Wt.(kg.)	22	1.16	0.32	0.56	1.68

pp = post-partum, Wt. = weight, VFD = variable foraging demand,

*missing data on one subject

### ANOVA-RM for CSF CRF versus plasma cortisol concentrations

**Fig 2:**
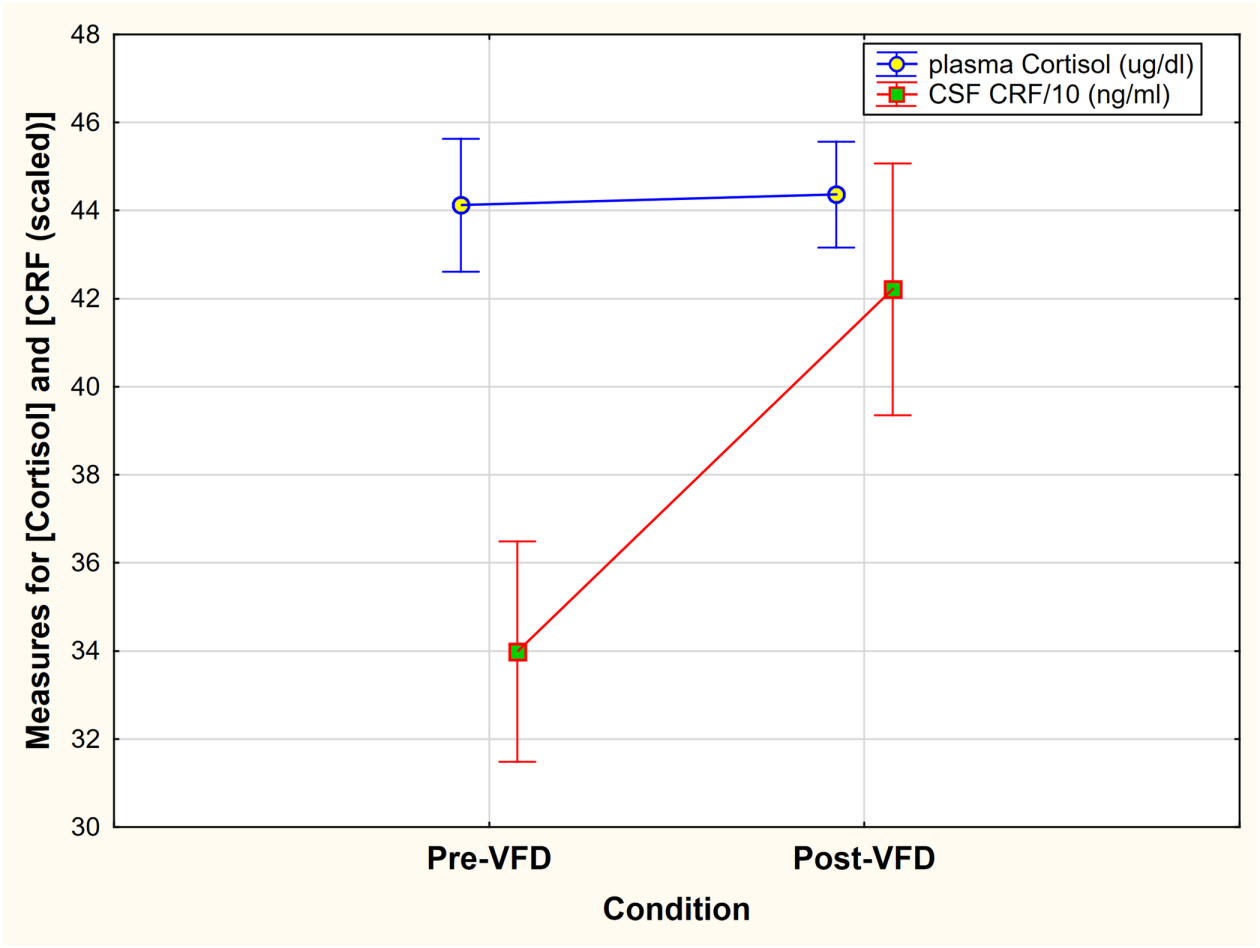
Group mean changes of CSF CRF concentrations and plasma Cortisol concentrations pre- and post-VFD exposure. ANOVA-RM revealed a Measure*Condition effect [F_(1,21)_ = 4.74, p = 0.04]. CSF CRF was scaled by a factor of 0.1 to approximate cortisol unit range. A significant increase in CRF group mean change and no group mean change in cortisol after VFD exposure was observed. Vertical bars denote standard errors.

To validate observations of the data we employed an ANOVA-RM and scaled CSF CRF concentrations by dividing by 10 to approximate plasma cortisol range. A Measure*Condition effect was noted [F_(1,21)_ = 4.74, p = 0.04]. While CRF increased significantly in response to VFD (Fig 2), no response was observed for plasma cortisol. The ANOVA-RM only provides data for mean changes so we proceeded to examine individual changes in HPA axis response in comparison to CSF CRF concentrations.

### Plasma cortisol response to VFD exposure

In a longitudinal analysis, pre-VFD plasma cortisol [mean (SD) = 44.12 (7.09) ug/dl] did not differ significantly from post-VFD cortisol [mean (SD) = 44.36 (5.61) ug/dl; t-value = -0.11; df = 21, p = 0.91] without statistically significant reductions in the variance of the post-VFD mean, which would be expected should there be a regression to the mean effect. The effect was not dependent on pen allocation [repeated measures x pen code: F_(1,20)_ = 0.59, p = 0.45]. The mean group change was -0.24 ug/dl with a mean % change of zero. The mean of individual cortisol change was 7.86 ug/dl (sd = 6.48) with a minimum Δ of– 19.5 and maximum Δ of 24.5 ug/dl with the % individual Δ mean over pre-VFD baseline = 19.8%. Thus, although the group mean of cortisol did not change, the mean shift of cortisol in response to VFD exposure from baseline for each subject was ~ 20%, indicating active HPA axis modulation during VFD. The correlation between pre-VFD and post- VFD cortisol was not significant (r = -0.24; p = 0.26; N = 22). By contrast, pre-VFD cortisol strongly correlated with Δ cortisol (r = −0.84, p = 0.0001, N = 22) (Fig 2) and Δ cortisol strongly predicted post-VFD cortisol (r = 0.73, p ˂ 0.001, N = 22). In general, subjects with high baseline cortisol, which was associated with our “vulnerability index” exhibited cortisol decreases. By contrast, larger, more dominant females started out with relatively low cortisol and showed plasma cortisol elevations.

### CSF CRF response to VFD exposure

VFD-exposure led to an increase in CSF CRF concentrations, validating that the model produced an allostatic overload. A significant increase in pre-to post-VFD CRF concentrations was observed [pre-VFD mean (SD) = 339.83 (117.40) pg/ml to post-VFD mean (SD) = 422.13 (134.08) pg/ml; t-value = -2.52; df = 21, p = 0.019]. Δ CSF CRF mean (SD) was 82.30 pg/ml (152.97) (post minus pre-VFD) with a minimum change of -214 pg/ml and maximum change of 458.47 pg/ml. The % Δ of the group mean over baseline was 24.2% whereas the % of the mean of each individual’s Δ (absolute values) divided by baseline was 56.8% (see Tables 3 and 4). The correlation between pre- and post-VFD CRF was not significant (r = 0.26; p = .23; N = 22). By contrast, pre-VFD CRF correlated inversely with Δ CRF (r = −0.53, p = 0.01, N = 22) and Δ CRF predicted post-VFD CRF (r = 0.67, p ˂ 0.01, N = 22).

**Table 3:** Relationship of maternal independent variable to Z-score transformed plasma cortisol and CSF CRF change (Δ = Post—Pre) to VFD exposure.

Maternal Variables	z-score of Δ cortisol	z-score of Δ CRF
Maternal Age (years)	0.34, p = 0.12	-0.18, p = 0.42
Maternal Weight (kg)	**0.58, p = 0.004** ^A^	-0.35 p = 0.10 ^A^
Pre-VFD Plasma Cortisol (ug/dl)	**-0.84, p = 0.001** ^B^	-0.09, p = 0.69^B^
Pre-VFD CSF CRF (pg/ml)	**-0.43, p = 0.045**	**-0.53, p = 0.010**
Maternal-infant Distance	**0.47. p = 0.030**	0.36, p = 0.10
Maternal Social Rank	**0.55, p = 0.007**^C^	-0.16, p = 0.46^C^

^A:^ Repeated Measures x Maternal Weight (kg): F_(1,20_) = 21.25; p = 0.0002 (low weight = ↓cort

^B:^ Repeated Measures x Maternal pre-VFD cortisol (ug/dl): F_(1,20_) = 9.63; p = 0.006 (high pre-VFD cort = ↓ cort)

^C;^ Repeated Measures x Social Rank: F_(1,20_) = 5.88; p = 0.024 (low social rank = ↓cort).

**Table 4:** Relationship of infant independent variable to Z-score transformed plasma cortisol and CSF CRF change (Δ = Post—Pre) to VFD exposure infant variables.

Infant Variables	z-score of Δ cortisol	z-score of Δ CRF
Infant age at VFD onset* (days)	0.39, p = 0.076	0.19, p = 0.39
Infant weight at VFD onset (kg)	**0.49, p = 0.020**^A^	-0.06, p = 0.76^A^
Infant sex (M = 1)	-0.18, p = 0.41	**0.43, p = 0.043**

^A:^ Repeated Measures x Infant Weight (kg): F_(1,20_) = 4.35; p = 0.05 (low infant weight = ↓cort)

*data on one subject not available, VFD = variable foraging demand, CSF = cerebrospinal fluid, CRF = corticotropin releasing-factor

Results of the 2x2 non-parametric table testing, as previously described, revealed a significantly reduced proportion of group mean percentage change/baseline versus percentage individual change mean/baseline for cortisol compared with CRF (Fisher exact p, two-tailed, N = 22, p = 0.002).

### Correlational analyses

#### Maternal variables

**Fig 3:**
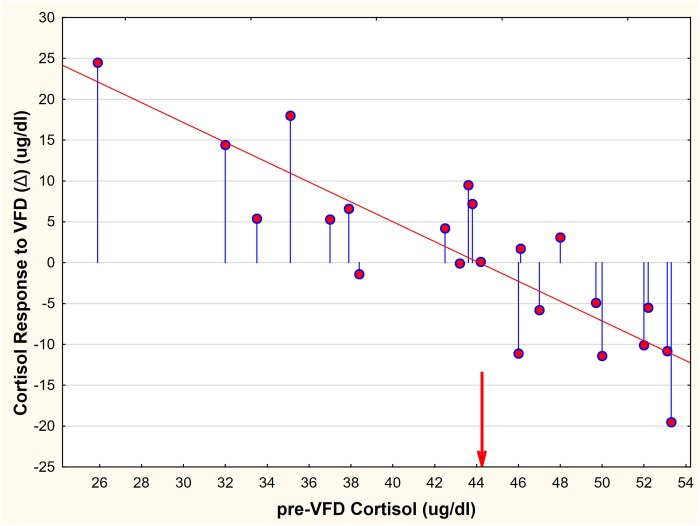
The relationship between maternal Pre-VFD cortisol and Δ cortisol (Post minus Pre-VFD) in response to maternal variable foraging demand. The red arrow indicates the maternal pre-VFD and post-VFD plasma cortisol mean (44.36) which was unchanged despite exposure to VFD. The blue lines represent distance from no change (Δ = 0). The red line represents the regression line [r = -0.84, p = 0.0001, n = 22).

Neither z-scores for Δ cortisol (z Δ cort) nor Δ CRF (z Δ CRF) correlated with maternal age. However maternal weight correlated positively with z Δ cort but not with z Δ CRF. These data implied that heavier, relatively advantaged mothers increased plasma cortisol in response to VFD exposure whereas lighter, hence generally vulnerable, subjects showed reduced cortisol (Tables 3 and 4). The cortisol versus CRF correlations were significantly distinguishable from each other implying that z Δ CRF was significantly less affected by maternal weight than was z Δ cort. Similarly, for social rank, z Δ cort correlated inversely, suggesting that mothers with dominant social rank exhibited relative cortisol increases whereas subordinates showed cortisol decreases. No effects were evident between social rank and z Δ CRF. Pre-VFD plasma cortisol correlated strongly with z Δ cort in an inverse manner, implying that subjects with relatively low HPA axis activation exhibited relative increases in cortisol whereas the converse was the case for subjects who had relative HPA axis activation pre-VFD (Fig 3). However, z pre-VFD cortisol did not relate to z Δ CRF and the two correlations were statistically distinguishable suggesting that the CRF system was functionally disassociated from the HPA axis in the context of maternal VFD exposure.

**Fig 4:**
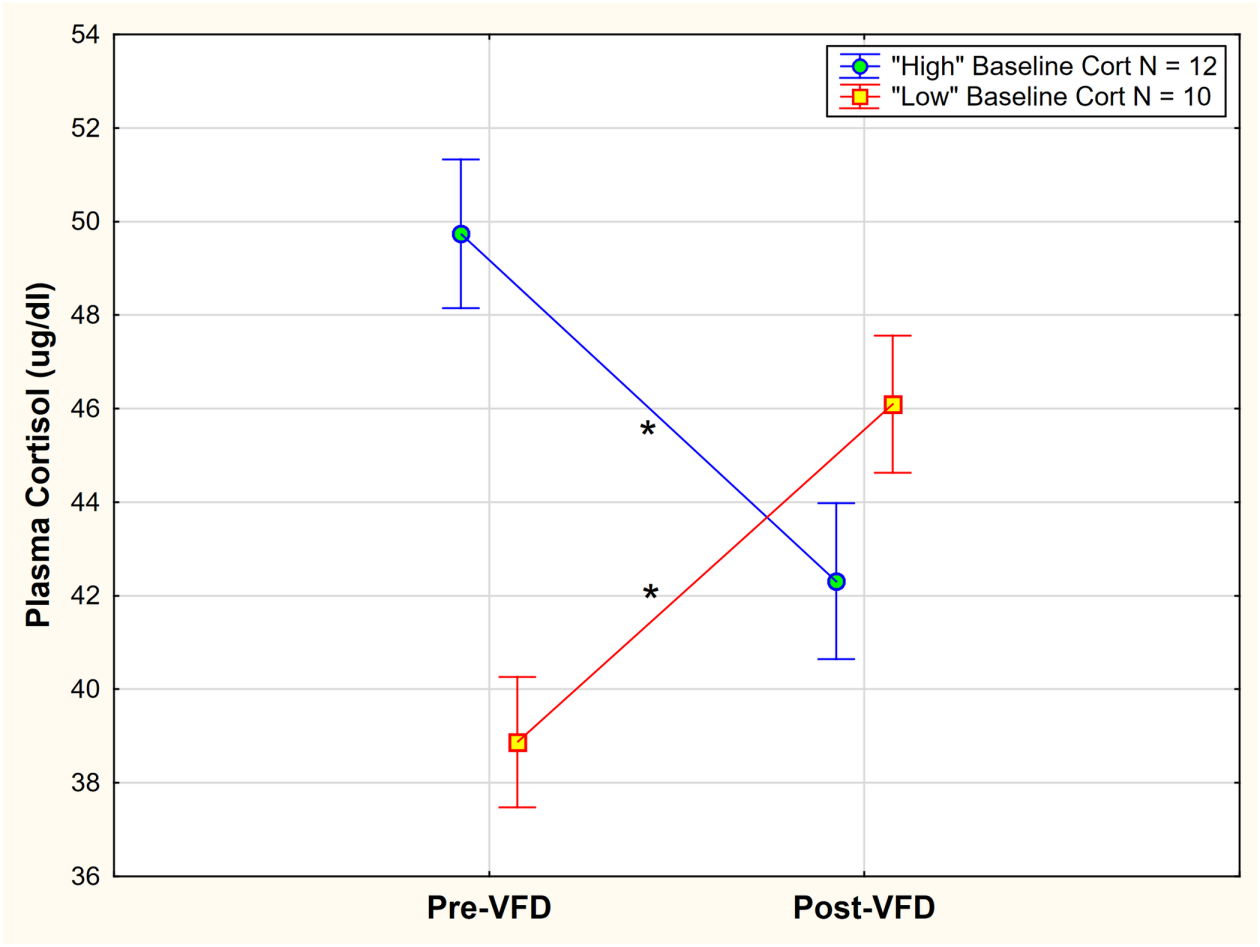
“High” versus “Low” baseline cortisol grouping and effects on plasma cortisol change in response to VFD exposure. A significant pre-VFD cortisol grouping x repeated measures interactive effect was noted [F_(1,20_) = 20.36; p = 0.0002, partial η^2^ = 0.54, 3.8 x greater than a large effect size] such that the “high” pre-VFD cortisol group exhibited cortisol decreases whereas the “low” cortisol VFD group showed cortisol increases. Vertical bars indicate ± standard errors. * indicates NK posthoc testing with p < 0.05.

Maternal VFD exposure, therefore, appeared to reverse HPA axis function depending on baseline cortisol. To statistically buttress the “HPA axis inversion” observation, pre-VFD cortisol values were coded by those above the mean cortisol value versus values below the mean. A GLM was then performed with “high” versus “low” pre-VFD cortisol code as the categorical variable and pre-and post-VFD cortisol as the repeated measure. A significant pre-VFD cortisol grouping x repeated measures interactive effect was noted [F_(1,20_) = 20.36; p = 0.0002, partial η^2^ = 0.54, 3.8 x greater than a large effect size] (Fig 4) such that the “high” pre-VFD cortisol group exhibited cortisol decreases [mean (SE) = 49.74 (1.54) to mean (SE) = 42.31 (1.71); N = 10, Newman-Keuls (NK) post-hoc testing p < 0.05] whereas the “low” cortisol VFD group showed cortisol increases [mean (SE) = 39.44 (1.41) to mean (SE) = 46.07 (1.56); N = 12, NK post-hoc testing p < 0.05]. Results were obtained while controlling for infant age. Although the “low” cortisol group exhibited numerically higher post-VFD cortisol levels (46.07) versus the ‘high” cortisol group post-VFD exposure (42.31), post-hoc testing indicated that the difference only achieved trend significance (NK post-hoc testing (p < 0.1).

#### Relationship of independent variables to HPA axis versus CSF CRF response

The relationship between pre-VFD cortisol and z Δ cort was significantly stronger than that between pre-VFD cortisol and z Δ CRF (which was not significant). Of note pre-VFD CSF CRF concentrations inversely predicted both z Δ cort and z Δ CRF implying relatively low pre-VFD CSF CRF concentrations predicted relative increases of both cortisol and CRF. Thus, one point of intersection between the central CRF system and the HPA axis is evident at the pre-VFD stage. Maternal infant-distance correlated positively with z Δ cort and not z Δ CRF implying that greater distance between mother and infant was associated with greater increases in maternal cortisol whereas reduced dyadic distance was accompanied by cortisol decreases.

#### Infant variables

Infant age at the onset of VFD exposure marginally predicted maternal z Δ cort in a direct fashion, and no effect was noted for z Δ CRF. However, infant weight at onset of VFD exposure directly predicted maternal z Δ cort such that relatively low infant weight, a vulnerability factor during periods of unpredictable food availability, was associated with relative reductions in maternal HPA axis activity and *vice versa*. This effect was noted to be more evident for z Δ cort in comparison to z Δ CRF. Infant sex had no effect on z Δ cort but mothers with female infants demonstrated CSF CRF concentration increases in response to VFD exposure to a greater degree than mothers with male infants.

Of note, neither z Δ CRF (r = 0.19, N = 22, p = 0.38) nor z Δ cort (r—0.20, N = 22, p– 0.39) correlated with the sequence of pen used for the four pens utilized in the current study.

#### Cluster analysis and “dyadic vulnerability” index

**Fig 5:**
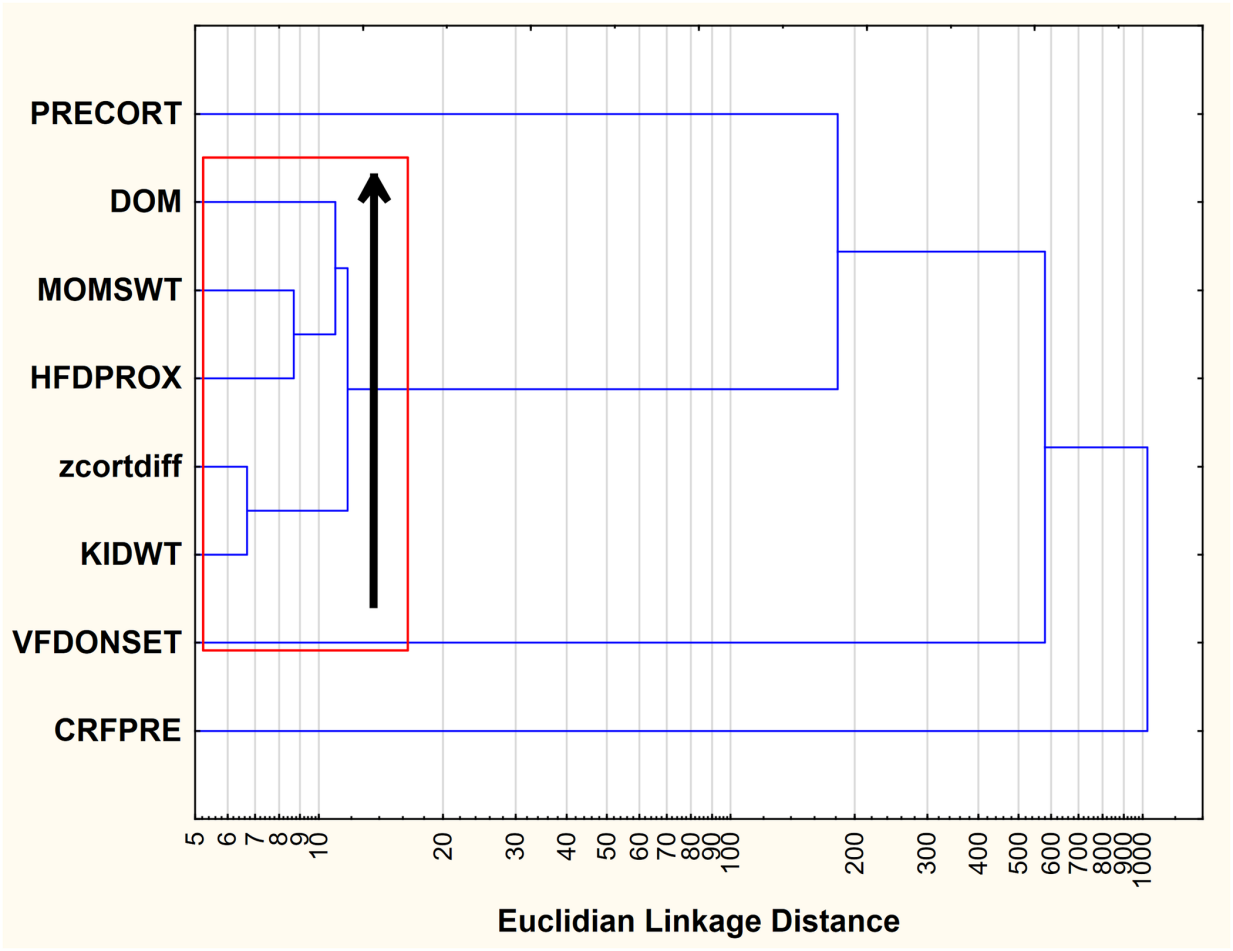
Cluster analysis of eight factors potentially related to core variable associated with a dyadic vulnerability index. The cluster analysis demonstrates the factors that promote maternal cortisol decrements include lighter, more subordinate mothers with lighter infants at the time of VFD onset, with reduced maternal-infant dyadic distance suggesting an association between socioecological vulnerability and cortisol suppression. The cluster analysis suggests a “core” of vulnerability variables (in red outlined area) that are closely associated. The black arrow indicates that core variables were each less than 50 Euclidian Linkage Units from each other. However, given that the VFD group mean cortisol remains stable, cortisol decrements are evidently exquisitely matched by cortisol increments in relatively advantaged, yet equivalently challenged, dyads, hence the concept of social allostasis. Abbreviations: PRECORT = maternal pre-VFD cortisol (ug/dl), DOM, = social rank score, MOMSWT = maternal weight measured prior to VFD exposure, HFDPROX = maternal-infant proximity aggregated score obtained from the final high foraging demand two week epoch, z cortdiff = standardized z-score of post—pre-VFD cortisol in response to maternal VFD, KIDWT = infant weight at the time of onset of VFD exposure (kg), VFDONSET = age in days at onset of VFD, CRFPRE = maternal CSF CRF concentrations (pg, ml) obtained prior to VFD exposure.

Examination of the cluster analysis (Fig 5) revealed a tight grouping of “core” dyadic vulnerability variables. The core variables were each, using a post hoc cutoff, less than 50 Euclidian Linkage Units from each other. Variables entered into the cluster analysis included maternal rank status (low rank = ↓cortisol), maternal weight (low weight = ↓cortisol), dyadic proximity during maternal high foraging demand (proximity = ↓cortisol), infant weight (low weight = ↓cortisol) and maternal cortisol response to VFD exposure (high baseline = ↓cortisol). Pre-VFD cortisol is closely associated but not included in the core grouping. Infant age at the onset of VFD exposure and pre-VFD CSF CRF concentrations also appear relevant but are > 50 Euclidian Linkage Units compared to the core variables. We therefore created a "dyadic vulnerability" index that included the sum of a) z-score converted maternal weight (inverted), b) maternal social rank, c) infant weight at VFD onset (inverted) and d) dyadic proximity (inverted) during the final high foraging demand two-week phase of the VFD procedure. The final HFD phase was selected for study as that two-week period representing the greatest cumulative dyadic stress incurred by the VFD exposure paradigm.

**Fig 6:**
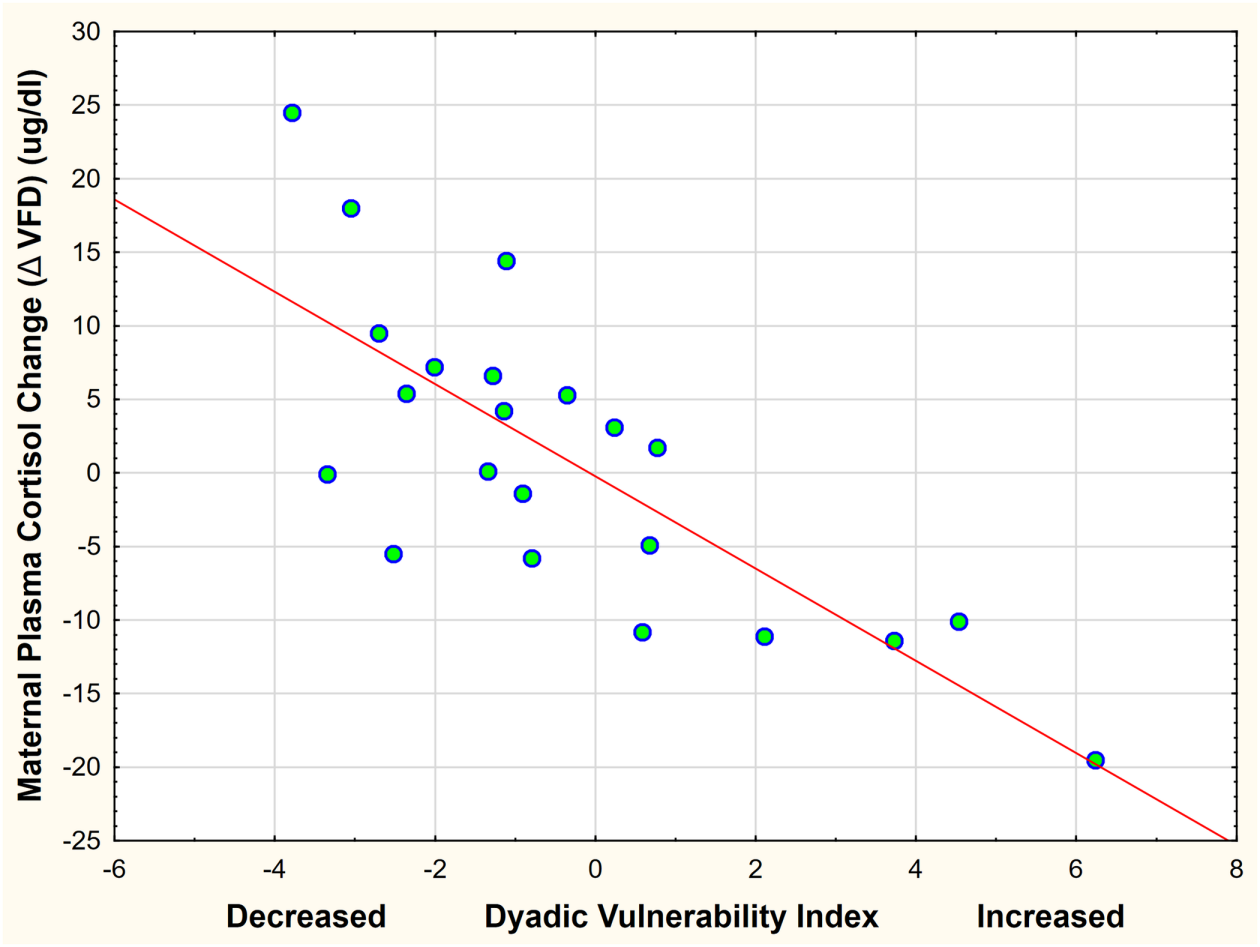
Cortisol response to VFD exposure in relationship to the “vulnerability index”. The vulnerability index inversely predicted cortisol change [F_(1,19)_ = 27.97, p < 0.0001; partial η2 = 0.59] (an effect size over four factors greater than a large effect size. Infant sex played no significant role in the relationship [F_(1,19)_ = 0.07, p = 0.78]. The red line represents a linear regression (r = -0.77; p <0.001; r^2^ = 0.60).

A general linear model was performed using the “Dyadic Vulnerability Index” as the dependent variable, infant sex as a control categorical and plasma cortisol change (post-VFD—pre-VFD) in response to VFD exposure. The vulnerability index inversely predicted cortisol change [F_(1,19)_ = 27.97, p < 0.0001; partial η^2^ = 0.59; (Fig 6); an effect size over four factors greater than a large effect size]. Infant sex played no significant role in the latter relationship [F_(1,19)_ = 0.07, p = 0.78]. We also examined to what degree the “vulnerability index” was independent of pre-VFD cortisol levels. The index, controlling for infant sex, accounted for 41% of the variance of pre-VFD cortisol levels [Adjusted R^2^ = 0.41; F_(2;19)_ = 8.33; p 0.0025] suggesting that 59% of the index’s variance was independent of maternal pre-VFD cortisol levels.

#### Cross-sectional analysis

**Fig 7:**
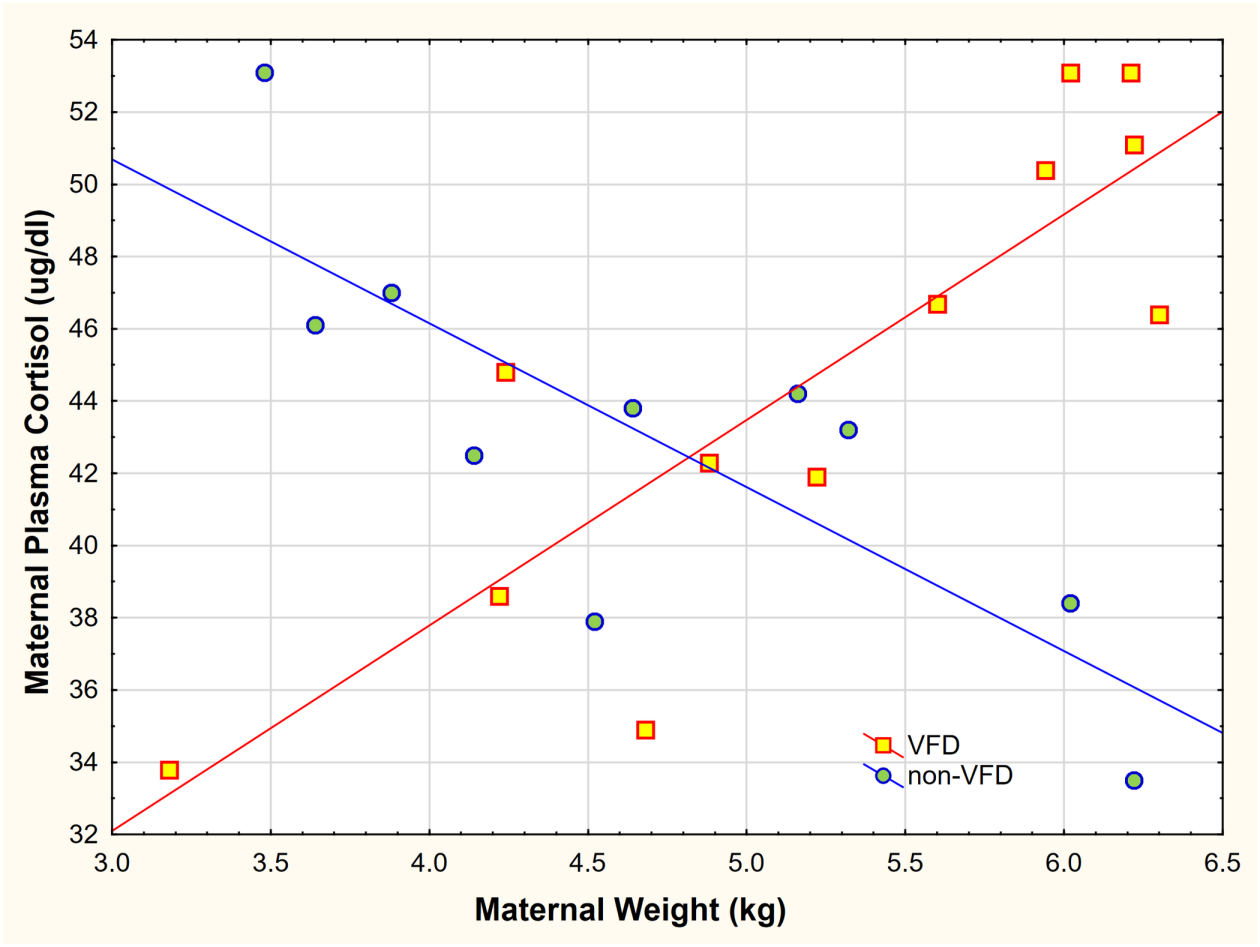
Effect of maternal body mass on maternal plasma cortisol as a function of variable foraging demand exposure. There was a general effect of increased cortisol level in the VFD-exposed group in comparison to the non-VFD group [F _(1; 19)_ = 27.72; p < 0.001], an effect absent when not controlling for body weight. A marked maternal weight by VFD exposure interaction was noted [F _(1; 19)_ = 29.53; p < 0.001]. A positive correlation (red line represents linear regression curve) was observed between maternal weight and maternal cortisol level for the VFD exposed group (r = 0.85, N = 12, p < 0.001) versus a negative correlation (blue line represents linear regression curve) in the non-VFD exposed group (r = -0.79, N = 10, p = 0.006).

For the cross-sectional analysis (Fig 7), the relationship between maternal plasma cortisol level and maternal body mass as a function of VFD exposure was examined in a factorial design fashion. Subjects were divided into their respective VFD- and non-VFD exposed groups. To counter the notion that our findings represented a non-specific time effect we analyzed the early and late VFD onset groups. The groups were equivalent for maternal and infant variables, except for infant age at the point of onset of VFD exposure [21]. There was a general effect of increased cortisol level in the VFD exposed group in comparison to the non-exposed group [F _(1; 19)_ = 27.72; p < 0.001] but this effect was not significant when body weight was controlled for in the model [F_(1; 21)_ = 0.26; p = 0.61]. A marked maternal weight by VFD exposure interaction was noted [F_(1; 19)_ = 29.53; p < 0.001; partial η^2^ = 0.60; (Fig 7); (an effect size over four factors greater than a large effect size)]. In the VFD-exposed group a positive correlation was observed between maternal weight and maternal cortisol level (r = 0.85, N = 12, p < 0.001) compared to a negative correlation in the non-exposed group (r = -0.79, N = 10, p = 0.006). Effects were unchanged when controlling for infant age at the onset of VFD or infant sex. Thus, this analysis counters the view that the “inversion” phenomenon of the HPA axis in response to VFD exposure is a “regression to the mean effect” or a non-specific time effect.

## Discussion

Variable Foraging Demand (VFD) is typically utilized as an experimental model for examining the behavioral and neurobiological sequelae of early life stress in grown *offspring* exposed to a socioecological stressor [14, 44]. In the current study, however, we used VFD as a model of *maternal* allostasis [18], wherein we analyzed maternal HPA axis alterations after socioecological instability on both an individual and at a group level. Our findings indicate that although CSF CRF concentrations are increased in response to VFD exposure, mean post-VFD cortisol is unchanged by 16 weeks of the VFD stressor (Fig 2). However, the within-group distribution of maternal cortisol post-VFD exposure values was significantly altered. Consistent with studies of previous unstable baboon social hierarchies where high levels of cortisol levels are observed in the dominant subjects [45], relative increases in cortisol levels were observed in dominant mothers, whereas relative suppression of plasma cortisol was observed in subordinate mothers. These data indicate that 1) social rank plays a significant role in determining the directionality and magnitude of the individual's allostatic response to VFD and 2) allostatic mechanisms also function beyond the level of the individual to maintain group homeostasis of cortisol levels for the social group, even when controlling for pen sequence.

### Within-subject analysis

“Dominants” pre-VFD began with relatively low cortisol and showed increases in response to VFD exposure while the subordinates started relatively high and lowered their cortisol after VFD. One possible hypothesis is that, under conditions of VFD, the dominant animals may be more calorically impacted by the foraging unpredictability, hence the cortisol increments relative to the subordinates as they muster the energy to monopolize resources. On the other hand, subordinates exhibit lower cortisol, which can provide a number of significant evolutionary adaptations when exposed to allostatic forms of stress: 1) minimizing glucose utilization and oxidative stress when food accessibility is restricted [30]; 2) promoting increased immunity for wound healing and prevention of infection if injured in a hierarchical encounter with a dominant mother; and 3) increasing sympathetic nervous system output, due to a lack of glucocorticoid dampening effects [46, 30].

Although pre-VFD cortisol levels were not associated with social rank, they strongly predicted the magnitude and directionality of the cortisol response. Subjects with relatively low cortisol pre-VFD increased cortisol levels whereas subjects with high pre-VFD plasma cortisol values exhibited reduced cortisol post-VFD and intermediate cortisol values remained relatively unchanged (Fig 4). Overall there was a broad range of cortisol differences in response to VFD exposure from a +24.5ng/ml increase to a -19.5 ng/ml cortisol decrease—a 44 ng/ml variation (post minus pre-VFD cortisol) in maternal glucocorticoid responsivity, the latter variation approximating mean cortisol values pre-VFD (44.12 μg/dl).

As can be seen in the cluster analysis (Fig 5), factors that promote maternal cortisol decrements include smaller, more subordinate mothers with smaller infants at the time of VFD onset, with reduced maternal-infant dyadic distance suggesting an association between socioecological vulnerability and cortisol suppression. The cluster analysis proposes a “core” of vulnerability variables that are closely associated and are accompanied by cortisol decrements. However, given that the VFD group mean cortisol remains stable, cortisol decrements are evidently exquisitely matched by cortisol increments in relatively advantaged, yet equivalently challenged, dyads, hence the concept of social allostasis. The evidence that relatively “advantaged” dyads are equivalently affected is that we have not been able to establish any biobehavioral variable in grown offspring that relates to dyadic “status” (see below) [21].

Using the “vulnerability index” comprising the z-score converted sum of the four “core” variables visualized on Fig 5 provides clear evidence of the inverse parametric relationship between heightening dyadic vulnerability and cortisol lowering responses to VFD exposure (Fig 6). Seen from the alternate vantage, relative increases in dyadic “advantage” is directly associated with increases in maternal cortisol. Moreover, we indicate that ~ 60% of the “vulnerability index” is independent of maternal pre-VFD cortisol values, a powerful determinant of Δ cortisol itself. As indicated previously [47], virtually none of our analyses have shown that ‘dyadic vulnerability”, or its components, predicts offspring phenotype yet the group mean may be affected versus controls not exposed to VFD. The latter observation indicates that compromise and de-prioritization of maternal care extend across the range of the “vulnerability index.” Consistent with the theme of the paper, each mother’s neuroendocrine physiology has to focus on regulating her own HPA axis response so as to complement the notion of a net zero group change. This process occurs in tandem with the mothers regulating dyadic distance in tandem with their social rank (>80% of the variance), thereby deprioritizing maternal care of the infant [21].

Maternal cortisol change was not predicted by maternal CSF CRF response to VFD, but relatively high pre-VFD CSF CRF predicted cortisol decreases and vice versa. Moreover, that elevated CSF CRF prior to VFD predicted cortisol decrements suggests that cortisol suppression may relate, in part, to trait levels of relative activation of the central CRF system. These data are consistent with parallel work in our laboratory indicating that juvenile CSF CRF elevations are directly associated with increases in adult monocyte glucocorticoid receptor mRNA expression, which would tend to be associated with cortisol reductions (see Appendix).

Staggering the timing of VFD onset by spreading the age of infants at the time of VFD onset demonstrated that maternal plasma cortisol levels and CSF CRF concentrations prior to VFD did not vary as a function of the duration (in days) of the post-partum period (pp) prior to VFD onset [18]. The sizeable range of infant age at the point of VFD onset allowed us to capture the effects of relative infant immaturity (vulnerability) on maternal glucocorticoid response. Low infant body weight at the time of VFD onset (a close proxy of infant age) represented an important source of variance for maternal cortisol decrements, and was included in the “vulnerability index.” Moreover, by staggering onset of VFD for infants [18], we were able to compare matched VFD-exposed to non-VFD exposed mothers and demonstrate that the relationship between maternal body mass and cortisol is fully inverted [partial η^2^ effect size = 0.6, which is over four times higher than a larger effect size (0.14)] as a function of VFD exposure (Fig 7). Relatively high body mass under VFD exposure triggers HPA axis activation whereas, at baseline, heavier animals maintain relatively low HPA activation. The opposite is true of the relatively light females where at baseline relatively high cortisol is maintained, but VFD exposure promotes cortisol decrements. The polar responses of the HPA axis suggest a physiological template [48] that facilitates each mother’s biobehavioral adaptation as an individual, yet each mother is also tethered to a common mandate for group HPA axis homeostasis [49].

Our results are consistent with the findings of a recent meta-analysis where it was noted that male and female primates differ in their respective relationships between glucocorticoid production and social status as a result of differences in social behavior and associated costs involved in acquiring and maintaining rank [50]. More specifically, dominant males typically produce more glucocorticoids than subordinate males since their rank is achieved by violent turnovers of existing hierarchy, and competition over access to mates. On the other hand, subordinate females produce more glucocorticoids than dominant females in hierarchies where rank is achieved via subtle aggressive and affiliative interactions, and competition is over access to quality food resources. In our study, we found that subordinate females also exhibited higher cortisol levels than dominant females at baseline, but a reversal of this pattern occurred after VFD exposure.

It must be noted that the results of the current study are in direct contrast to the phenomenon of “regression to the mean” [51], wherein “post-intervention values change no more than is required to approximate the overall mean more closely.” In the current study, cortisol difference values, particularly in those mothers with markedly high and low initial levels, are equidistant from the mean for both pre- and post-VFD values, thus comprising an observed pattern of *relative symmetrical inversion* (Fig 4). Since these pre-VFD maternal cortisol levels “cross” the mean to an equivalent but opposite degree over the 16 weeks of VFD exposure, the data suggest that the VFD stressor exerts a distinct and predictable response for each mother by virtue of her pre-VFD trait characteristics in addition to her dyadic response. Moreover, regression to the mean would be accompanied by a significant reduction in variance measures over time, despite an unchanged mean, which is not the case.

### Overall group analysis

The absence of mean change in cortisol in the face of large within group changes, accompanied by an overall increase in CSF CRF concentrations from pre-to post-VFD, indicate that homeostatic adjustments serve to maintain overall group HPA axis function within a narrow range. The current data are consistent with recent results, where overall mean glutamate levels were maintained within a narrow range in response to VFD, despite the marked within-subject changes [52] (see Appendix).

To our knowledge, the current study is amongst the first to demonstrate that “allostasis” may occur at a group level where the mean cortisol level of mothers who underwent a chronic stressor, in this case, VFD exposure, remained unchanged despite marked within-subject changes. In a previous study of male cynomolgus monkeys, subordinate subjects exhibited significantly higher cortisol concentrations on initial social housing, but by the end of 12 weeks, dominants were found to have higher cortisol and testosterone. However, those monkeys were not subjected to experimentally controlled conditions of stress in the 12-week period, and mean cortisol concentrations of the entire group before and after 12 weeks were not addressed [47].

Our demonstration of allostasis functioning at the group level suggests that all subjects who underwent the VFD stressor exhibited, to some degree, physiological synchronization suggesting functioning as a single mammalian entity or organism. In individual organisms, chronic elevations of glucocorticoids can lead to a multitude of effects, including weight gain [53], suppressed immune system functioning [54], and impaired adaptive responses to acute stress [50]. It remains to be determined what advantage, if any, would be gained through the maintenance of mean cortisol stability in the face of the VFD form of socioecological stress. Certainly, since caloric intake in the VFD paradigm is kept steady, albeit, with uncertainty, the “social allostasis” phenomenon would enhance the ability of the social group, as a single entity, to cope with resource stress for prolonged time periods through individualized redistribution of a fixed caloric amount.

Consistent with the current theme of the paper “biologists have long recognized that adaptations of a higher than individual level of organization must also be an important source of adaptation and that such selection must be recognized to account for adaptations that work for the benefit of groups instead of individuals” [55]. According to Williams, group adaptations may arise purely by statistical chance, a possibility in the current study. However, Gardner and Grafen have provided a hypothesis as to how individuals contribute to a “superorganism” and propose a formalized theoretical model for group adaptation [27]. The current paper provides support for the view that nonhuman primate HPA axis function may well serve as a “superorganism mandate” particularly in mother rearing offspring under conditions of food uncertainty stress. The question is raised—under what circumstances can the concept of adaptation be applied to groups, rather than individuals [56]. Gardner and Grafen [27] are recognized for bringing a level of “mathematical precision” to the issue of group adaptation by applying Grafen's ‘formal Darwinism’ project,’ which provides a general framework for understanding the concept of adaptation for groups. The concept of adaptive “optimization” is captured by an ‘objective function’ that mathematically maps an agent's phenotype to its ‘fitness’ (e.g., maternal HPA axis adaptation under reproductive viability stress). The authors invoke the notion that should “an agent achieve the maximum value of this function, they are said to ‘behave optimally’.” We would argue that the group adaptation to resist group HPA axis alterations captures the “maximum value of the function.”

### Limitations

Several caveats regarding the current data should be considered. The first issue relates to the limitations of a single pre- and post-VFD sampling of plasma and CSF. Because we elected not to intervene during VFD so as not to disturb the experimental process, we are unable to assess the rate and pattern of maternal cortisol secretion that together comprise the divergent forms of cortisol response observed in response to VFD exposure. Moreover, more detailed HPA axis studies, such as the dexamethasone suppression tests with or without CRF administration [57, 58] may have shed light on pathophysiological mechanisms in the current study [59]. Our main goal, however, was to obtain measures that were reflective of ambient glucocorticoid function within the context of the VFD stressor.

Another limitation was the longitudinal design yet the creation of a VFD non-exposed control group was feasible by virtue of the staggered design [19]. The cross-sectional analysis clearly demonstrates that VFD exposure significantly alters the relationship between maternal weight and HPA axis response in comparison to non-exposed subjects. These data refute the notion that the results of the study may be attributable solely to a time effect.

Another caveat is that we fail to address potential physiological mechanisms that may mediate social allostasis. Additional studies are clearly required, but proof of concept has been provided. A final limitation may be the questionable relevance of “closed” social systems to the complexity of human social interactions. We argue that humans may, despite living in “open” social systems, shrink their social template to create internal “closed” social hierarchies, particularly when unstable, and resources become unpredictable [60].

## Supporting information

S1 Table Please see supporting information for full data set in Microsoft Excel format. (XLSX)
